# Diaphragm Ultrasound in Cardiac Surgery: State of the Art

**DOI:** 10.3390/medicines9010005

**Published:** 2022-01-11

**Authors:** Abdallah Fayssoil, Nicolas Mansencal, Lee S. Nguyen, David Orlikowski, Hélène Prigent, Jean Bergounioux, Djillali Annane, Frédéric Lofaso

**Affiliations:** 1 Echo Lab, CHU de Raymond-Poincaré, AP-HP, Boulevard Raymond Poincaré, 92380 Garches, France; 2 INSERM U1179, END-ICAP, Université de Versailles-Saint Quentin, University of Paris-Saclay, 78180 Montigny-le-Bretonneux, France; helene.prigent@aphp.fr (H.P.); f.lofaso@aphp.fr (F.L.); 3 Raymond Poincaré Hospital, AP-HP, Boulevard Raymond Poincaré, 92380 Garches, France; 4 Centre de Référence des Cardiomyopathies et des Troubles du Rythme Cardiaque Héréditaires ou Rares, Department of Cardiology, Ambroise Paré Hospital, Assistance Publique-Hôpitaux de Paris, AP-HP, Université de Versailles-Saint Quentin, 92100 Boulogne, France; nicolas.mansencal@aphp.fr; 5 INSERM U-1018, CESP, Épidémiologie Clinique, 94807 Villejuif, France; 6 Service de Médecine Intensive-Réanimation, Hôpital Cochin, AP-HP, Centre, 27 rue du Faubourg Saint-Jacques, 75014 Paris, France; nguyen.lee@icloud.com; 7 France Research and Innovation Department, CMC Ambroise Paré, RICAP, 27 bd Victor Hugo, 92200 Neuilly-sur-Seine, France; 8 Service de Réanimation Médicale, CHU Raymond Poincaré, AP-HP, Université de Versailles Saint Quentin en Yvelines, 92380 Garches, France; David.ORLIKOWSKI@aphp.fr; 9 Centre d’Investigation Clinique et Innovation Technologique CIC 14.29, INSERM, 92380 Garches, France; 10 Service de Physiologie et Explorations Fonctionnelles, GH Paris Ile de France Ouest—Site Raymond Poincaré—AP-HP, 92380 Garches, France; 11 Pediatric Neurology and ICU, Assistance Publique-Hôpitaux de Paris, Hôpital Raymond-Poincaré, 92380 Garches, France; jean.bergounioux@aphp.fr; 12 Laboratory Infection and Inflammation, Department of Critical Care, Raymond Poincaré Hospital (AP-HP), U1173, Faculty of Health Science Simone Veil, Université de Versailles-Saint Quentin, University Paris Saclay, INSERM, FHU SEPSIS, RHU RECORDS, 78180 Montigny-le-Bretonneux, France; djillali.annane@aphp.fr

**Keywords:** cardiac surgery, diaphragm ultrasound, phrenic nerve, cardiac ICU

## Abstract

In cardiac surgery, patients are at risk of phrenic nerve injury, which leads to diaphragm dysfunction and acute respiratory failure. Diaphragm dysfunction (DD) is relatively frequent in cardiac surgery and particularly affects patients after coronary artery bypass graft. The onset of DD affects patients’ prognosis in term of weaning from mechanical ventilation and hospital length of stay. The authors present a narrative review about diaphragm physiology, techniques used to assess diaphragm function, and the clinical application of diaphragm ultrasound in patients undergoing cardiac surgery.

## 1. Introduction

Diaphragm is the most important inspiratory muscle and is innerved by the phrenic nerve. In cardiac surgery, patients are at risk of phrenic nerve injury, which leads to diaphragm dysfunction (DD) [1]. The onset of DD is a serious outcome, affecting morbidity and length of hospital stay [1,2,3,4,5]. This is particularly a pejorative burden in patients with previous pulmonary diseases. In fact, after cardiac surgery, pulmonary complications are frequent, with an incidence reaching 30% in adults [2,6]. In addition, the incidence of DD, after cardiac surgery may reach 75% [7,8,9]. Many factors are involved in the onset of respiratory complications after cardiac surgery, namely, general anesthesia, invasive mechanical ventilation, damage of the surfactant, presence of sternotomy and cardiopulmonary bypass. Diaphragm dysfunction may be related to perioperative phrenic nerve injury, either during harvesting of the internal mammary artery (IMA) or in relation to cold injury caused by local hypothermia [4,10]. Diaphragm ultrasound can be performed at the bedside to assess diaphragm function. This technique may help physicians to manage patients in the context of postoperative weaning trials and to stratify patients regarding respiratory prognosis.

This review aims to report the physiological aspects of diaphragm muscle and phrenic nerve, the radiological and non-radiological techniques used to assess diaphragm function, and the clinical applications of diaphragm ultrasound in cardiac surgery.

## 2. The Diaphragm

The diaphragm is the main inspiratory muscle, contributing to 60–70% of the total ventilation at rest. Respiratory system elastic recoil refers to the respiratory system’s intrinsic tendency to deflate following inflation. The diaphragm is a dome-shaped fibro-muscular structure with a central tendon and a cylindroid muscular portion, inserted within the inner rib cage and attached to the xiphisternum and the lumbar vertebrae. Physiologically, there is a rhythmic contraction of the diaphragm against elastic and resistive forces. During expiration, the diaphragm returns progressively to a relaxation state position determined by the balance between chest wall and lung recoil forces [11]. During inspiration, the diaphragm shortens and moves caudally in a piston-like manner, with an increase of the abdominal pressure and a decrease of the pleural pressure, leading to a decrease of the alveolar pressure, below atmospheric pressure. This generates airflow into the lungs against a resistance, following the principles stated by Ohm’s Law [12]. Accordingly, the transpulmonary pressure, i.e., the difference between alveolar pressure and intrapleural pressure, is a key determinant of lung volume. During inspiration, gastric pressure increases as the diaphragmatic muscle contracts, descends, and displaces the abdominal content downward and outward [13]. In the meantime, there is an outward displacement of the lower ribs, firstly due to the insertional force applied by the contraction of the fibers that exert a direct, cranially oriented force on these ribs [14]. The second mechanism, also called the “appositional force,” is due to the transmission of abdominal pressure to the lower rib cage in the apposition zone [14].

Diaphragm strength affects the inspiratory lung volume directly. In addition, with diaphragm displacement, the scalene, parasternal, and external intercostal muscles are activated during tidal breathing, preventing the downward displacement of the upper ribcage due to the negative pleural pressure caused by the diaphragm displacement [13,14,15]. In a patient breathing steadily, the expiratory process is passive, depending on the respiratory system recoil [13]. The abdominal muscles may be solicited with contraction during expiration in patients with respiratory failure, followed by relaxation providing assistance to inspiration if the volume at the end of expiration decreases below the equilibrium volume, i.e., the functional residual capacity.

The neural control of the diaphragm relies on the phrenic nerve. After originating from C3–C5 (anterior horn), it crosses the scalenus anterior muscle. The right phrenic nerve, at the apex of the thorax, crosses the right IMA, runs along the right side of the superior vena cava, then goes along the pericardium over the right cardiac cavities before reaching the diaphragm [8]. The left phrenic nerve, after crossing the scalenus muscle, runs along the left subclavian artery and behind the thoracic duct, before crossing the left IMA and descending in the thorax, closer to the pericardium, adjacent to the left ventricle, and finally reaches the diaphragm. Since the phrenic nerves are closer to the pericardium, they are vulnerable to the cooling methods used to protect the myocardium during cardiac surgery. In addition, since the phrenic nerves are closer to the IMA in the apical region of the chest, they are also vulnerable during the harvesting phase [8].

## 3. Traditional Techniques Used to Assess the Diaphragm Function

Historically, diaphragm function analysis relies on trans-diaphragmatic pressure measurement or fluoroscopy in radiology. The latter requires ionizing radiation and specific devices, and patients need to be transported to the radiology department, which is a major drawback for patients hospitalized in the Intensive Care Unit (ICU). Other exams can be performed at the bedside. Pulmonary functional tests (FPT) can be used to measure the maximal inspiratory pressure (MIP) [16]. MIP is an index of the inspiratory muscles strength that includes the diaphragm and the intercostal and accessory inspiratory muscles. It depends on lung volume and is classically measured at residual volume, although the recoil of the chest wall may be a confounding element. Hence, this technique is not specific to measure the diaphragm strength, considering that the ribcage muscles can generate inspiratory pressures at the airway opening without a significant diaphragm contribution. In addition, this technique is volitional and depends on the patient’s motivation, cooperation, and ability. In the context of post-cardiac surgery, it is not usual to perform this test, since the patient may be in a painful situation (i.e., sternotomy, Redon drain). The maximal sniff nasal inspiratory pressure (SNIP) is another parameter used to assess respiratory muscles’ strength at the bedside. SNIP is measured using a pressure transducer connected to a catheter localized in the nostril, at the functional residual capacity (FRC) level. SNIP is not classically recorded in ICU after surgery, as it is not reliable for intubated/tracheostomized patients. *Delta* pulmonary vital capacity (VC) can analyze diaphragm function. *Delta* VC is the change of VC from the upright to the supine position. A decrease in *delta* VC is an index of diaphragm dysfunction [16]. The ratio of maximal expiratory pressure (MEP) to maximal inspiratory pressure (MEP/MIP) can also be used as an index of diaphragm dysfunction. In fact, patients with diaphragm dysfunction but with preserved expiratory muscle function have a decreased MIP but a normal MEP. The MEP/MIP ratio can be used as an alternative to assess diaphragm function in patients that can tolerate the supine position.

The measurement of the trans-diaphragmatic pressure (*pdi*) is the reference test to assess diaphragm strength, although it is an invasive technique. It requires a gastric balloon catheter and an esophageal balloon catheter, providing the measurement of gastric and esophageal pressures, respectively. The *pdi* is equal to the gastric pressure minus the esophageal pressure. It can be obtained in patients during volitional maneuvers such as maximal inspiratory effort (pdi *max*) at FRC, during a sniff maneuver (pdi *sniff*) [17], or during a magnetic stimulation (non-volitional maneuver). Normal *pdi* values swing during these maneuvers, depending on sex, size, initial volume of the respiratory system, and body position. High values exclude diaphragm weakness. Normal values range from 100 to 150 cm H₂O [17]. The Gilbert index, which is the ratio between *delta* gastric pressure and delta *pdi*, can also be used to determine the contribution of the diaphragm during inspiration, after cardiac surgery. A high value is associated with a great contribution of the diaphragm muscle during inspiration. Twitch mouth pressure is a non-invasive and non-volitional test that can be used to assess diaphragm function in non-intubated patients [18]. This measurement requires a flanged mouthpiece connected to a pneumotachograph, which is in turn connected to a differential pressure transducer [18]. For this test, magnetic phrenic nerve stimulation is necessary. However, these techniques require expertise and are time-consuming. In addition, the stimulation of phrenic nerves cannot be performed in patients with implanted cardiac devices (pacemakers or defibrillators).

## 4. Diaphragm Ultrasound Technique

Diaphragm ultrasound is a noninvasive radiological exam that can be performed at the bedside. To assess the diaphragm, the patient is positioned in the semi-recumbent position, ideally on spontaneous breathing. Two approaches are classically used to assess diaphragm function.

### 4.1. The Subcostal Approach

**Figure 1:**
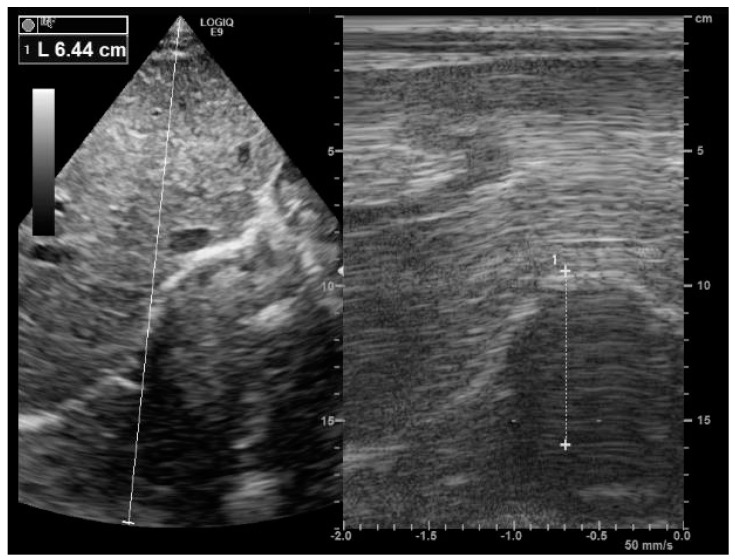
Right diaphragm ultrasound. Note the normal inspiratory motion (64 mm) of the hemidiaphragm from the subcostal view. After the visualization of the right hemidiaphragm (bright line) using a B-mode (image on the left), an M mode was applied (image on the right) to record diaphragm motion.

**Figure 2:**
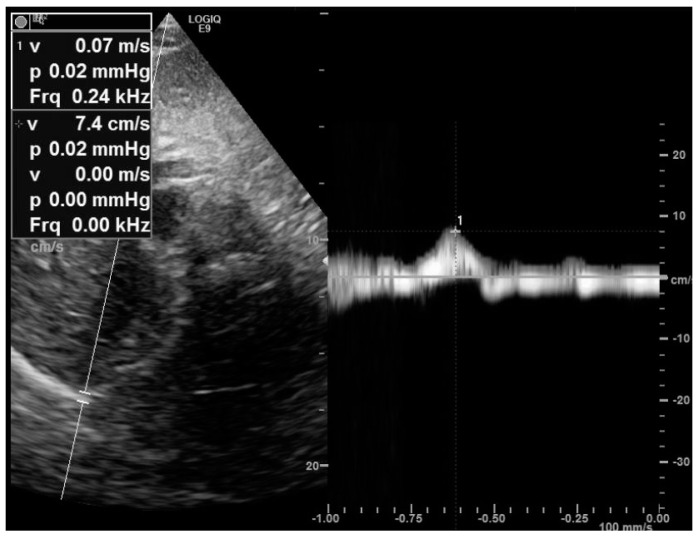
Measurement of the right peak sniff tissue Doppler imaging velocity from the subcostal view. The diaphragm velocity was recorded during a sniff maneuver. Here is a reduced peak sniff velocity (7 cm/s) in a patient with muscular dystrophy.

Diaphragm motion is measured at the mid-clavicular line or at the antero-axillary line in the patient. For right hemi-diaphragm analysis, the liver is used as an acoustic window, whereas the spleen is used for left hemidiaphragm analysis. The operator visualizes the right diaphragm as a bright line covering the liver. The normal diaphragm moves toward the probe (supplementary information), and we record an upward motion from M mode tracing (Figure 1). Normal values were published, with high intra- and inter-reproducibility [19]. During quiet breathing, the normal diaphragm motion was reported to at 13.4 ± 1.8 mm [19]. In the study by Boussuges et al. [19], normal values of diaphragm motion during a deep inspiration were 70 ± 6 mm for males and at 57 ± 1 mm for females. During tidal breathing, diaphragm weakness is defined by a diaphragm inspiratory motion less than 10–15 mm [20,21,22]. While the analysis of right hemidiaphragm motion is easily performed in routine practice, left hemidiaphragm motion is harder to assess, since during the inspiration phase, the descending lung and the bowel interposition decrease the visibility of diaphragm mobility. Finally, tissue Doppler imaging coupled to a sniff maneuver can be used to assess diaphragm function (Figure 2), and normal values have been published [23].

### 4.2. The Apposition Zone Approach

**Figure 3:**
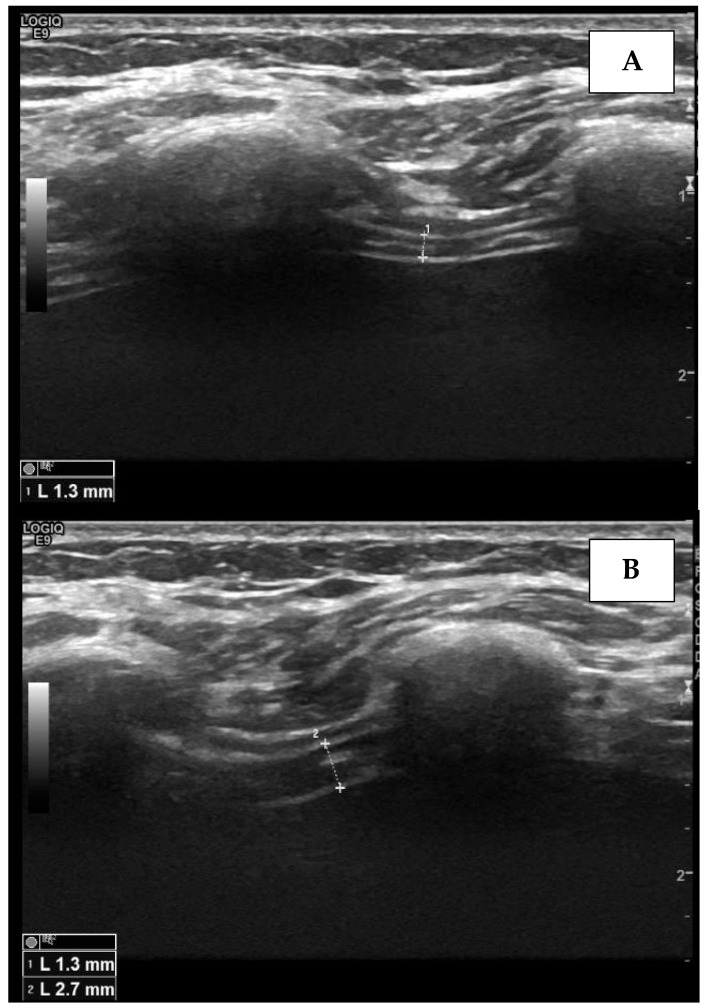
Measurement of the right diaphragm thickness (dotted line) in the end-expiratory phase (**A**) and end-inspiratory phase (**B**), using ultrasound. The diaphragm is visualized as a hypo-echogenic central layer surrounded by two hyper-echogenic lines, namely, the pleural line and the peritoneum.

The apposition zone is the area of the chest within the abdomen that reaches the lower ribcage. To assess the diaphragm from the apposition zone, it is essential to use a linear high-frequency probe. For the echographic procedure, the probe has to be positioned on the mid-axillary line or on the antero-axillary line, perpendicular to the zone of apposition, in the 8th to 11th intercostal space. The diaphragm can be visualized as a hypo-echogenic central layer surrounded by two hyper-echogenic layers, namely, the pleural line and the peritoneum. Figure 3 shows diaphragm thickness measurement from the apposition zone using ultrasound at end-expiration and at end-inspiration, providing diaphragm thickening.

Normal values have been published [20]. The normal diaphragm end-expiratory thickness is more than 1.7 mm [24]. Diaphragm thickness may vary with gender and body composition. In a study that included 150 healthy subjects with a mean body mass index (BMI) of 27.9 ± 4.7 kg/m², the normal right expiratory diaphragm thickness was 3.8 ± 1.5 mm for males and 2.7 ± 1 mm for females [20]. In the study by Ueki et al. [25], the normal diaphragm thickness was 1.7 ± 0.2 mm at rest, reaching 4.5 ± 0.9 mm at total lung capacity (TLC). In another study that included 109 healthy participants with a mean BMI of 24.1 ± 3.6 kg/m², Carrillo-Esper et al. [26] reported a mean diaphragm thickness of 1.9 ± 0.4 mm for men and 1.4 ± 0.03 mm for women. From the end-expiratory diaphragm thickness and end-inspiratory diaphragm thickness, it is possible to calculate the diaphragm thickening fraction (TF) = (end-inspiratory thickness-end-expiratory thickness)/end-expiratory thickness). Diaphragm TF is reproducible [27], and a diaphragm TF value below 20% is a marker of diaphragm paresis [17] and diaphragm paralysis [21]. Diaphragm dysfunction can be defined by a diaphragm TF < 20–36% [21]. Table 1 summarizes the normal values of diaphragm ultrasound parameters

## 5. Diaphragm Dysfunction in ICU

Diaphragm dysfunction is common in ICU and affects prognosis, respiratory outcomes, length of hospital stay, and weaning process [4]. In ICU, the prevalence of diaphragm dysfunction at admission was reported to be up to 64% by Demoule et al. [35], while in patients on weaning trials, the prevalence reached 23% to 80% [22,24,36]. The mechanisms of diaphragm dysfunction are multiple and include mechanical ventilation-induced diaphragm disuse, sepsis, use of drugs (propofol, steroid, neuromuscular blockers), and excessive loading [37].

The consequences of diaphragm dysfunction are severe, resulting in an increase of mechanical ventilation duration [22,36], risk of re-intubation, and mortality [35]. Hence, monitoring diaphragm function with ultrasound may be useful. In fact, diaphragm ultrasound parameters *cut-off* to predict weaning trial success or failure from mechanical ventilation have been published [38]. Thus, Pirompanich et al. [39] reported a right diaphragmatic TF *cut-off* ≥ 26% to predict successful weaning. Ferrari et al. [40] reported a *cut-off* value of diaphragm TF > 36%, for predicting success weaning in ICU patients on spontaneous breathing. In the study by DiNino et al. [24], a diaphragm TF ≥ 30% could predict extubation success.

Other authors focused on diaphragm excursion. In fact, the magnitude of diaphragm excursion during inspiration can predict the success of a weaning trial [41,42]. Diaphragm excursion is considered abnormal if it is lower than 10–14 mm [19]. In the study by Kim et al. [22], a diaphragm weakness defined by diaphragm inspiratory motion <10 mm was associated with weaning features. The diaphragm inspiratory motion *cut-off* to predict successful extubation was 11 mm, using a T tube trial, in the study by Jiang et al. [43]. A right inspiratory motion *cut-off* > 10 mm was reported by Jung-Wan Yoo et al. [44] to predict successful extubation. Finally, a combined index has been proposed to predict weaning trial success, namely, the diaphragm excursion time index, which is the product of diaphragm excursion and inspiratory time [45]. In the study by Palkar et al. [45], during a spontaneous breathing trial, the diaphragmatic excursion time index was 2.42 ± 1.55 cm.s in the group with successful weaning trial versus 1.64 ± 1.19 cm.s (*p* < 0.03) in the group with failure weaning trial. Spadaro et al. [46] proposed another index, that is, the diaphragmatic RSBI (respiratory rate/diaphragmatic motion), to predict weaning failure. A diaphragmatic RSBI *cut-off* > 1.3 can predict weaning failure, whereas a *cut-off* diaphragm motion ≤ 14 mm can predict weaning failure [46]. Table 2 summarizes the *cut-off* values of diaphragmatic ultrasound parameters used to predict weaning outcome in ICU patients.

## 6. Diaphragm Dysfunction in Cardiac Surgery

Diaphragm dysfunction is frequent after cardiac surgery, particularly after coronary artery bypass graft (CABG) [1] and in patients with diabetes mellitus [47]. In addition, obesity and arterial hypertension may be risk factors for post-operative diaphragmatic dysfunction [4]. Preoperative inspiratory muscle weakness may predict the postoperative duration of mechanical ventilation in patients undergoing cardiac surgery [5]. Clinically, patients with bilateral diaphragm paralysis disclose orthopnea, rapid shallow breathing, and thoraco-abdominal paradoxus in the supine position [48]. The abdominal paradoxus, an inward motion of the abdomen during inspiration, is related to a flaccid diaphragm, drawn up by the contraction of the intercostal muscles during inspiration. Physical examination may find an increase of the respiratory rate, the utilization of accessory inspiratory muscles, and a contraction of the abdominal expiratory muscles. On physical exam, the analysis of ribcage expansion as well as of abdomen displacement may indirectly provide information regarding diaphragm features.

In practice, we distinguish two situations, in the context of phrenic features after cardiac surgery:

1. The management of patients in cardiac ICU after surgery, in the context of a weaning trial
2. The management of patients with moderate symptoms who may reveal diaphragm features in cardiac rehabilitation unit.

In cardiac ICU, DD after cardiac surgery is frequent. In the study by Moury et al. [10], DD, defined by a diaphragm TF < 20% during a spontaneous breathing trial, was present with an incidence reaching 75%. Length of surgery, cross-clamp time, and cardio-pulmonary bypass time negatively affect diaphragm thickening [10]. In addition, propofol and remifentanil were associated with a decrease of diaphragm TF [10]. In the study by Diehl et al. [3], that included 13 patients with DD, after cardiac surgery, the authors reported high morbidity and mortality, with the occurrence of nosocomial pneumonia, cardiorespiratory arrest after early extubation, prolonged mechanical ventilation, and death. In a recent French study that included 3577 patients, the presence of DD after cardiac surgery was associated with the risk of post-operative pneumonia, non-invasive ventilation, and invasive ventilation and an increase of ICU hospital length of stay [4]. Table 3 summarizes studies that assessed diaphragm function and clinical outcomes of adult patients after cardiac surgery.

It is essential to provide risk stratification for patients undergoing cardiac surgery. Preoperative diaphragm function may be used as a prognostic tool. Indeed, preoperative diaphragmatic function has been reported to be associated with pulmonary outcomes after surgery [2]. A recent study reported an increase of post-operative pulmonary complications that include atelectasis, pneumonia, and prolonged mechanical ventilation in patients with a pre-operative diaphragm TF below 38% [2]. Hence, diaphragm ultrasound may help assess operative risk, in addition with the EuroSCORE II (European System for Cardiac Operative Risk Evaluation), in patients undergoing cardiac surgery [2].

## 7. Management of Diaphragmatic Dysfunction after Cardiac Surgery

**Figure 4:**
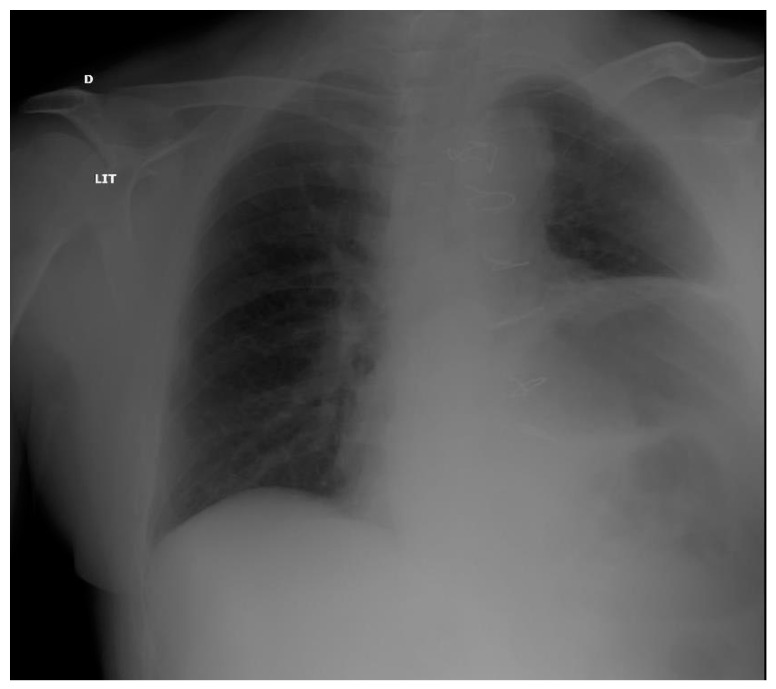
Chest X ray in a post-operative patient with diaphragm paralysis. Note ascension of the left diaphragm.

The diagnosis of diaphragmatic failure after a cardiac surgery relies on clinic, radiology, and blood gas exchange analyses. Blood gas exchange analysis may reveal acute respiratory acidosis. PFT cannot be performed immediately after sternotomy. Diaphragm elevation on chest X ray (Figure 4) may warrant specific exploration; however, it is not very specific (44%), as patients with pneumonia, atelectasis, and diaphragmatic eventration may also present this radiological sign [54]. Fluoroscopy can show classical diaphragm paradoxical motion during the sniff maneuver. The measurement of trans-diaphragmatic pressure provides clues for diaphragm paralysis, but this invasive technique is not routinely performed. A phrenic nerve electrophysiological study can be performed; however, this technique is not routinely performed in cardiac ICU. Furthermore, after cardiac surgery, patients are frequently equipped with temporary external pacemakers connected to epicardial electrodes, which confound electromyograms [17].

Conversely, diaphragm ultrasound can be done routinely in cardiac ICU after surgery. In patients with diaphragm dysfunction documented with ultrasound, spontaneous breathing trial (SBT) must be cautious, and CPAP (continuous positive airway pressure) can be proposed to support the respiratory status after extubation. The use of diaphragm ultrasound may reduce the time to extubation, according to a recent randomized trial [55]. In fact, using a *cut-off* of diaphragm TF ≥ 30% in ICU patients undergoing SBT, Mc Cool et al. [55] reported a significant reduction of the time from ultrasound to extubation in an interventional group in comparison with a group receiving usual care. Further studies in patients undergoing cardiac surgery are warranted in the post-operative period to determine the role of ultrasound in clinical practice.

## 8. Conclusions

Diaphragm is the main inspiratory muscle.

The assessment of diaphragm function relies on invasive and non-invasive tests.

Ultrasound can be used to assess diaphragm function. Diaphragm dysfunction is relatively frequent in patients after cardiac surgery. This feature affects patients’ prognosis. Performing diaphragm ultrasound may help to stratify and manage patients in the context of cardiac surgery.

## Figures and Tables

**Table 1:** Normal values of diaphragm parameters determined by ultrasound.

Author (Ref)	Year	*N*	Diaphragm Motion	Diaphragm Thickness	Diaphragm TF (%)	Diaphragm TDI or 2D Strain
Wait [28]	1989	10		2.2 ± 0.4 mm (FRC)		
Cohen [29]	1994	10	Deep inspiratory motion: 60 ± 7 mm			
Ueki [25]	1995	13		1.7 ± 0.2 mm (FRC) 4.5 ± 0.9 mm (TLC)		
Kantarci [30]	2004	164	Right DM: 49.2 ± 10.9 mm Left DM: 50.17 ± 11.7 mm			
Boussuges [19]	2009	210	QB: 9 mm (F) and 10 mm (M) DB: 37 mm (F) and 47 mm (M) Sniff echo: 16 mm (F) and 18 mm (M)			
Testa [31]	2011	40	QB: 18.4 ± 7.6 mm DB: 78.8 ± 13.3 mm			
Boon [20]	2013	150		Right: 3.3 ± 1 mm (FRC) Left: 3.4 ± 1.8 mm (FRC)		
Orde [32]	2016	50	Right: 49 ± 10 mm	Right: 2.4 ± 1 mm	Right TF: 45.1% ± 12%	Right diaphragm Strain: −40.3% ± 9%
Carrillo-Esper [26]	2016	109		Female: 1.4 ± 0.3 mm(FRC) Male: 1.9 ± 04 mm (FRC)		
Scarlata [33]	2018	100	QB: 17.6 ± 5.4 mm DB: 62 ± 15.5 mm			
Fayssoil [23]	2019	27	Right DB: 72 mm Left DB: 62 mm			Peak sniff TDI velocity: 13 cm/s (male) 12 cm/s (female)
Spiesshoefer [34]	2020	70	QB: 15.6 ± 5.3 mm DB: 80.2 ± 19.1 mm	1.9 ± 0.6 mm (FRC) 5.3 ± 1.8 mm (TLC)		

TF: thickening fraction; TDI: tissue Doppler imaging; FRC: functional residual capacity; TLC: total lung capacity; DM: diaphragm motion; QB: quiet breathing

**Table 2:** Diaphragm ultrasound parameters *cut-off* values for predicting success from mechanical ventilation weaning in ICU patients.

First Author (Ref)	*N* (Patients)	Diaphragm US Parameter *Cut-Off*	Sensibility	Specificity	*Reference* Test
Pirompanich [39]	34	TF ≥ 26%	96%	68%	RSBI
Dres [41]	76	TF > 26%	79%	73%	Twitch pressure using phrenic nerve stimulation
DiNino [24]	63	TF ≥ 30%	88%	71%	
Ferrari [40]	46	TF > 36%	82%	88%	RSBI
Yoo [44]	60	TF ≥ 30%	68.1%	61.5%	
Yoo [44]	60	DM > 10 mm	80.9%	69.2%	
Jiang [43]	55	DM > 11 mm	84.4%	82.6%	
Kim [22]	82	DM < 10 mm *	83% *	41% *	RSBI
Spadaro [42]	51	Diaphragmatic RSBI > 1.3 *	94%	64.7%	RSBI
Palkar [45]	73	Decrease of diaphragm ET index < 3.8% **	79.2%	75%	RSBI

US: ultrasound; ICU: intensive care unit. TF: diaphragm thickening fraction; RSBI: rapid shallow breathing index: respiratory rate/tidal volume. ET index: excursion time index = product of diaphragmatic motion and inspiratory time. *: to predict weaning failure. **: between assist-control ventilation and spontaneous breathing trial.

**Table 3:** Studies that analyzed diaphragm dysfunction and outcomes after cardiac surgery.

First Author (Ref)	Population (*N*)	Outcome	DD Diag	Prevalence Incidence DD/PNI after Cardiac Surgery	Factors Associated with DD	Prognosis in Patients with DD
Markand [49]	Cardiac surgery (44)	Phrenic nerve palsy after cardiac surgery	EPS	11% PNI		
Canbaz [50]	Cardiac surgery (78)	Effects on PNI of hypothermia, ice slush, and use of mammary artery harvesting	EPS	10.2% PNI	Hypothermia Ice slush	
Dimopoulou [9]	Cardiac surgery (63)		EPS	21% PNI	Ice slush	
Yamazaki [47]	CABG (200)	Incidence and factors associated with hemidiaphragm elevation after CABG	radiological study	14.5% hemi-diaphragm elevation after CABG	Diabetes and use of internal thoracic artery grafting are risk factors	
DeVita [51]	Cardiac surgery (92)	Incidence of phrenic neuropathy after cardiac surgery	radiological and EPS studies	Abnormal DM in 54% of patients with abnormal CR 57% PNI among patients with abnormal DM		
Merino-Ramirez [52]	CABG (94)	Incidence of phrenic neuropathy after CABG	EPS	16% PNI		
Bruni [1]	Cardiac surgery CABG 71% (100)	Rate of post-operative DD	TF < 20%	38%	Duration of cardiopulmonary bypass	Higher rate of difficult weaning, Longer ICU length of stay
Moury [10]	Cardiac surgery CABG 46% (100)	Diaphragm thickening during weaning	TF < 20%	75%	Length of surgery	
Tralhao [53]	(79)	Diaphragm US in patients with cardiac surgery	DM < 10 mm	36% at D2 after surgery		
Laghlam [4]	3577	Incidence, risk factors, and outcomes of patients with postoperative DD		7.6%	HTA Higher BMI CABG	Post-operative pneumonia Reintubation, ventilation, ICU hospital stay duration

CABG: coronary artery bypass graft; CR: chest radiography; Diag: diagnosis; D: day; DD: diaphragm dysfunction; DM: diaphragm motion; PNI: phrenic nerve injury; EPS: electrophysiological study of phrenic nerve; HTA: arterial hypertension; BMI: body mass index; ICU: intensive care unit.

## Supplementary Materials

The following supporting information can be downloaded at: https://www.mdpi.com/article/10.3390/medicines9010005/s1. Video S1: The video shows the normal diaphragmatic motion from the right subcostal view using a cardiac probe.
